# Comprehensive and Non-Destructive Sweet Corn Shelf-Life Prediction Using Near-Infrared (NIR) Spectroscopy Coupled with Multivariate Curve Resolution-Alternating Least Squares (MCR-ALS) Spectral Resolution

**DOI:** 10.3390/molecules31142512

**Published:** 2026-07-18

**Authors:** Sujitra Funsueb, Chanat Thanavanich, Chevaporn Chudoung, Phanaphon Jomnong, Parichat Theanjumpol, Sila Kittiwachana

**Affiliations:** 1 Department of Chemistry, Faculty of Science, Chiang Mai University, Chiang Mai 50200, Thailand; sujitra.funsueb@cmu.ac.th (S.F.); chanat.thanavanich@postgrad.manchester.ac.uk (C.T.); chevaporn_chu@cmu.ac.th (C.C.); 2 Office of Research Administration, Chiang Mai University, Chiang Mai 50200, Thailand; 3 Postharvest Technology Research Center, Faculty of Agriculture, Chiang Mai University, Chiang Mai 50200, Thailand; phanaphon.j@cmu.ac.th (P.J.); parichat.thean@cmu.ac.th (P.T.); 4 Postharvest Technology Innovation Center, Science Research and Innovation Promotion and Utilization Division, Office of the Ministry of Higher Education, Science, Research and Innovation, Bangkok 10400, Thailand; 5 Center of Excellence for Innovation in Chemistry, Faculty of Science, Chiang Mai University, Chiang Mai 50200, Thailand

**Keywords:** near-infrared (NIR) spectroscopy, comprehensive analysis, multivariate curve resolution-alternating least squares (MCR-ALS), multivariate accelerated shelf-life testing (MASLT), postharvest quality, kinetic degradation models

## Abstract

Accurate shelf-life prediction of perishable products remains challenging because quality deterioration involves multiple physicochemical changes that are not adequately captured by conventional univariate approaches. This study proposes a multivariate shelf-life prediction framework for sweet corn based on near-infrared (NIR) spectroscopy coupled with multivariate curve resolution–alternating least squares (MCR-ALS). NIR spectra were collected from sweet corn samples and analyzed using MCR-ALS to extract chemically interpretable concentration and spectral profiles. A total of 100 and 85 corn samples were used for model training and validation, respectively. The dominant MCR-ALS component showed strong correlations with total soluble solids, dry matter, and individual sugar contents (sucrose, glucose, and fructose), effectively describing the overall quality degradation process. Based on the zero-order kinetic model, the predicted shelf lives were 41.3, 11.0, and 8.9 days at 4, 13, and 25 °C, respectively. Arrhenius analysis of the MCR-ALS concentration profile yielded a temperature-dependent degradation rate with an activation energy of 54.05 kJ mol^−1^ (R^2^ = 0.8387). The practical applicability of the proposed framework was further examined using a separate harvest batch of sweet corn that underwent repeated non-destructive NIR measurements throughout storage. Overall, the proposed NIR–MCR-ALS framework provides a rapid, non-destructive, and chemically interpretable approach for shelf-life prediction and postharvest quality monitoring of perishable produce.

## 1. Introduction

Agricultural products, especially those that are highly perishable, deteriorate during storage and distribution. This deterioration results in quality degradation, rendering products unsuitable for consumption and potentially posing food-safety risks. The decline in quality is driven by physical, chemical, and microbiological processes influenced by temperature, humidity, and oxygen [1]. These factors determine shelf life, which refers to the duration over which a product remains acceptable in terms of sensory quality, nutritional value, and safety under given conditions [2]. Therefore, reliable and accurate shelf-life prediction is essential for minimizing postharvest losses, improving supply-chain management, and enhancing sustainability and profitability.

Sweet corn (*Zea mays* L. saccharata) is one of the most widely consumed vegetables worldwide. Its characteristic sweetness arises from a genetic mutation that suppresses the conversion of sugars to starch in the kernels [3]. However, its high sugar and water contents also make fresh sweet corn extremely perishable, requiring careful handling and accurate shelf-life determination to preserve quality [4]. Quality changes can be evaluated through external traits (color, weight loss, or texture) and internal traits for example, total soluble solids (TSS), dry matter (DM), and individual sugar concentrations, which are more reliable indicators of consumer quality and market value.

Traditionally, shelf-life estimation has been performed using accelerated shelf-life testing (ASLT), where degradation kinetics of quality attributes are individually modeled under elevated temperatures and extrapolated to normal storage conditions using the Arrhenius equation [5]. Parameters such as TSS, sugar content, firmness, and volatile compounds are often used as indicators [6,7]. Although straightforward, this univariate approach, based on a single quality parameter, often leads to inconsistent predictions and fails to capture the complexity of overall product deterioration [8,9].

Near-infrared (NIR) spectroscopy measures sample absorbance in the 700–2500 nm region, capturing overtone and combination bands arising from fundamental molecular vibrations. Its major advantages include rapid analysis, non-destructive measurement, and minimal sample preparation [10]. NIR spectroscopy has been widely used to assess physicochemical parameters such as TSS, titratable acidity, sugar content, and texture, in apricots [11], apples [12], desert ginseng [13], and corn [14]. However, the direct prediction of agricultural product shelf life using NIR spectroscopy remains challenging because postharvest degradation involves complex multivariate chemical changes reflected in NIR spectra, whereas degradation kinetics are typically modeled using a single quality attribute. Therefore, appropriate spectral preprocessing and chemometric analyses are required to extract meaningful information from NIR spectra for kinetic modeling.

Recently, several chemometric modellings, such as principal component analysis (PCA) and partial least squares (PLS) regression, have been used to capture major spectral variations and develop kinetic degradation models for shelf-life prediction of agricultural products [15,16]. More advanced multivariate accelerated shelf-life testing (MASLT) procedure integrates multiple quality attributes into a single predictive model derived from spectral data [17]. This approach allows the simultaneous evaluation of multiple quality traits; however, it requires a sufficient amount of quality-parameter data to simulate their behaviors.

Multivariate curve resolution–alternating least squares (MCR-ALS) is a powerful chemometric method that decomposes spectral data into concentration profiles and corresponding pure spectral signatures of underlying constituents [18]. As a bilinear resolution technique, MCR-ALS has been applied extensively in analytical chemistry [19], environmental analysis [20], pharmaceuticals [21], and food science [22]. For example, it has been used to resolve overlapping spectral contributions in pigment degradation [23], fermentation monitoring [24], and lipid oxidation [25]. Unlike PCA, which produces abstract principal components representing maximum variance, MCR-ALS yields interpretable concentration and spectral profiles directly related to chemical and physical phenomena in the samples. However, its potential for modeling kinetic degradation and predicting shelf life in postharvest crops has not yet been systematically explored.

Previous studies have primarily employed PCA for exploratory analysis and PLS regression for quantitative prediction of individual quality parameters. As summarized in Table 1, these chemometric methods have different objectives and provide complementary information. In contrast to PCA and PLS, which are primarily used for data exploration and quantitative prediction, respectively, MCR-ALS resolves the underlying chemical evolution during storage by decomposing the NIR spectra into chemically interpretable concentration and spectral profiles. These concentration profiles can subsequently be used as integrated quality indicators for kinetic degradation modeling and shelf-life prediction.

This study aimed to develop a non-destructive multivariate shelf-life prediction model for sweet corn using NIR spectroscopy coupled with MCR-ALS. In this approach, MCR-ALS resolves the NIR spectra of stored corn samples into chemically meaningful concentration profiles, which are subsequently employed to construct the kinetic degradation and the Arrhenius prediction models. The proposed method offers an integrated, rapid, and interpretable approach for the non-destructive prediction of postharvest quality and remaining shelf life in sweet corn, thereby facilitating improved storage management and reducing postharvest losses.

## 2. Results and Discussion

### 2.1. Changes in the Quality of Sweet Corn During the Storage

**Figure 1:**
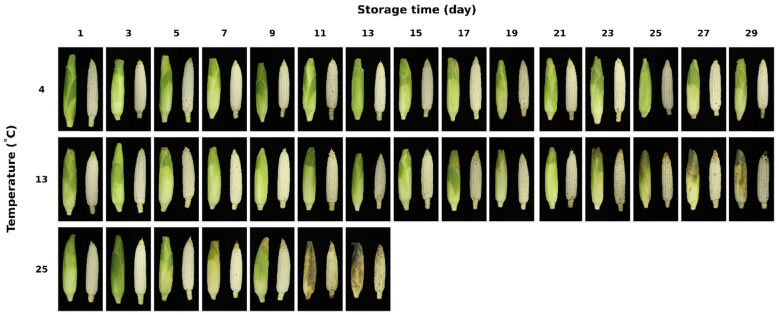
Appearance of sweet corn samples at different storage times and temperatures.

A collective photograph of the sweet corn samples during the storage period is shown in Figure 1. It is evident that the quality of sweet corn deteriorated as storage time increased. This decline was indicated by husk discoloration and browning, dented kernels, and visible mold growth. Storage temperature had a significant effect on the postharvest quality parameters of sweet corn (Table 2).

At 4 °C, the corn experienced minimal deterioration, maintaining green husks and remaining free of visible mold throughout storage. At 13 °C, deterioration became apparent, with husk discoloration observed by day 19. At 25 °C, the husk color turned brown as early as day 11, accompanied by visible mold growth. Consequently, storage and data collection for the samples at 25 °C were terminated after 13 days. As a result, the training set size decreased from 120 to 100 samples, while the test set size decreased from 105 to 85 samples.

**Figure 2:**
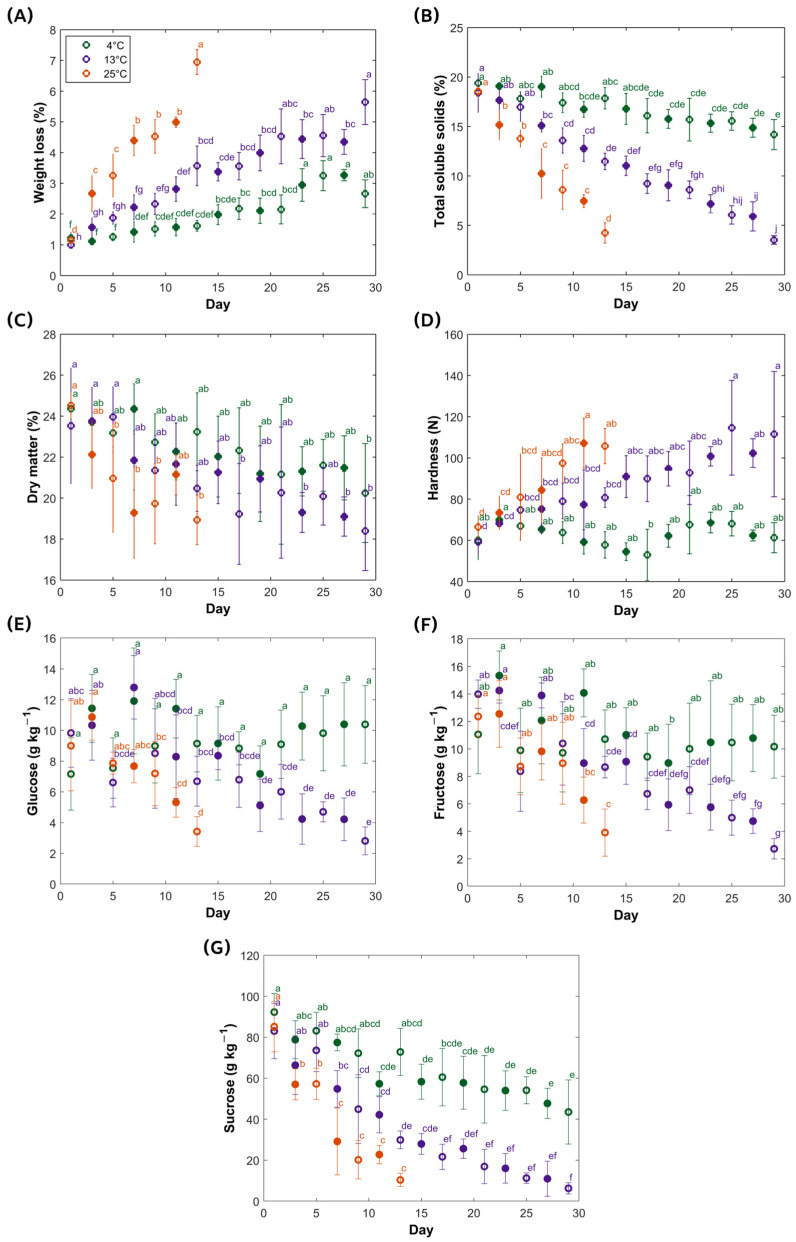
Changes in corn quality parameters during storage (**A**) weight loss, (**B**) total soluble solids, (**C**) dry matter, (**D**) hardness, (**E**) glucose, (**F**) fructose, and (**G**) sucrose. Different letters indicate significant differences among storage times at the same temperature (Tukey’s test following ANOVA, *p* < 0.05).

Quality changes were assessed using several parameters, including weight loss, DM, TSS, hardness, and sugar content (Table 2, Table S1, Figure 2). Weight loss increased significantly at 13 °C and 25 °C. TSS decreased with increasing storage temperature, particularly at 25 °C, where it declined from 18.54% on day 1 to 4.24% on day 13 (Figure 2B). DM also decreased during storage, from 24.52% to 18.93% for the corn stored at 25 °C (Figure 2C), mainly due to sugar conversion and respiration under elevated temperatures. Kernel hardness increased with storage time, consistent with moisture loss and pericarp thickening.

As shown in Figure 2E–G, fructose, glucose, and sucrose decreased rapidly at storage temperatures of 13 °C and 25 °C, with sucrose showing the most pronounced decline. Sucrose declined from 83.02 g kg^−1^ to 6.26 g kg^−1^ after 29 days at 13 °C, and from 85.18 to 10.35 g kg^−1^ after 13 days at 25 °C, greatly reducing sweetness and overall quality. In contrast, corn stored at 4 °C exhibited substantially slower sugar degradation, retaining 43.51 g kg^−1^ of sucrose on the final day of storage.

Overall, sugar contents declined with time, but the reduction at 4 °C was considerably less pronounced, indicating that low temperature slowed metabolic activity [26]. The more rapid decline in sugar content at higher storage temperatures can be attributed to increased respiration and metabolic activity, which accelerates the consumption of soluble sugars as energy sources. Elevated temperatures also promote the activities of carbohydrate-metabolizing enzymes, such as invertase and amylase, leading to the degradation and conversion of sugars during storage. Furthermore, higher temperatures favor microbial growth, which can further contribute to quality deterioration. Consequently, sweet corn stored at 4 °C retained higher sugar contents and sweetness than samples stored at 13 or 25 °C. Although the present study began measurements on Day 1 to monitor quality changes during storage, future studies should include measurements immediately after harvest (Day 0) to establish a baseline for the initial quality of sweet corn prior to storage.

### 2.2. Univariate Shelf-Life Prediction Based on Individual Quality-Related Parameters

The kinetic parameters of each corn quality parameter and the associated shelf-life estimations derived from zero-, first-, and second-order kinetic models are summarized in Table 3. In this study, the quality parameter data generally showed a better fit at 25 °C than at 13 °C. For instance, the R^2^ values for total soluble solids obtained from the zero-order kinetic model were 0.9873 and 0.9990 at 13 °C and 25 °C, respectively. Similarly, the first-order kinetic model for weight loss showed an increase in R^2^ values from 0.8912 to 0.9288 across these temperatures. The lower R^2^ values at 4 °C were consistent with the minimal experimental changes observed at lower storage temperatures (Figure 1 and Figure 2). As anticipated, the reaction rate constant (*k*) exhibited an increasing trend with rising temperature, reflecting faster quality degradation at higher storage temperatures.

In this experiment, corn samples stored at 25 °C on day 9 were selected as the shelf-life cut-off, as their quality remained acceptable, with green husks and no visible mold growth or kernel deterioration (Figure 1). Based on this criterion, the predicted shelf-life values varied depending on the quality parameter considered. Because the R^2^ values for the zero-order models were generally higher than those for the first- and second-order reactions, subsequent analysis focused on the zero-order kinetics. At 25 °C, the estimated shelf life ranged from 6.3 days for fructose to 11.6 days for hardness. At 13 °C, predictions extended from 11.7 days for fructose to 23.3 days for hardness. These results emphasize that the choice of quality parameter strongly influences shelf-life estimation, highlighting the difficulty of defining a single parameter to represent overall postharvest quality.

### 2.3. Exploratory Data Analysis of NIR Spectra

#### 2.3.1. NIR Spectra

**Figure 3:**
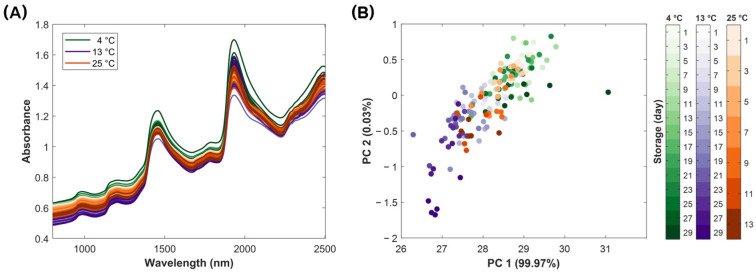
Preprocessed NIR spectra of the training and validation corn samples (**A**) and the corresponding PCA score plots (**B**). Each spectrum in (**A**) represents the average of ten scans acquired from an individual corn sample. The colors used for the spectra in (**A**) correspond to the color labels in the PCA score plots in (**B**).

Figure 3A presents the NIR spectra, after square-root scaling, acquired from the training and validation corn samples stored under different temperatures and storage durations. The prominent absorption bands were observed in the regions of 950–1050 nm, 1150–1250 nm, 1350–1650 nm, and 1850–2200 nm. The regions around 950–1050 nm and 1850–2200 nm correspond to the second overtone and combination bands of the O–H functional group, respectively. Meanwhile, the region of 1150–1250 nm is mainly attributed to the second overtone of the C–H stretching vibration in CH_2_ groups, whereas the region of 1350–1650 nm primarily contains the first overtone absorptions of O–H and C–H functional groups associated with water and carbohydrates. These absorption features are associated with the major chemical constituents of sweet corn, including water, sucrose, fructose, glucose, and starch [14,27].

Generally, NIR spectra represent overtone and combination vibrations of fundamental molecular absorptions in the infrared region. Consequently, the absorption bands are broad, highly overlapped, and relatively weak, making visual interpretation of spectral differences challenging. Nevertheless, as shown in Figure 3A, samples stored at 13 °C and particularly at 25 °C exhibited lower absorbance than those stored at 4 °C. This reduction is likely associated with accelerated sugar degradation, moisture loss through transpiration, and structural changes within the kernels during storage at elevated temperatures. Higher temperatures increase respiration and metabolic activity, leading to more rapid consumption of soluble sugars, while moisture loss and tissue structural changes alter the physical environment of O–H- and C–H-containing constituents. Consequently, these compositional and structural changes reduce the overall NIR absorbance compared with samples stored under refrigerated conditions.

#### 2.3.2. Principal Component Analysis of NIR Spectra

The differences among samples became more apparent in the PCA score plot (Figure 3B). All samples formed a single broad cluster, as they were all derived from corn, but variations associated with storage time and temperature were evident. For instance, corn stored at 4 °C showed higher PC1 and PC2 score values and was positioned in the upper-right region of the PCA space, whereas samples stored at 13 °C appeared in the lower-left region with lower PC1 and PC2 scores. Samples stored for longer periods tended to have lower PC2 score values. For example, corn ears stored at 4 °C for extended durations were located below the fresh samples, which are represented by the intense green symbols. The corn ears stored at 25 °C were mainly distributed between the 4 °C and 13 °C groups. In this case, most samples could be differentiated according to storage temperature, indicating that temperature had a more pronounced effect on the NIR spectral variation than storage time.

**Figure 4:**
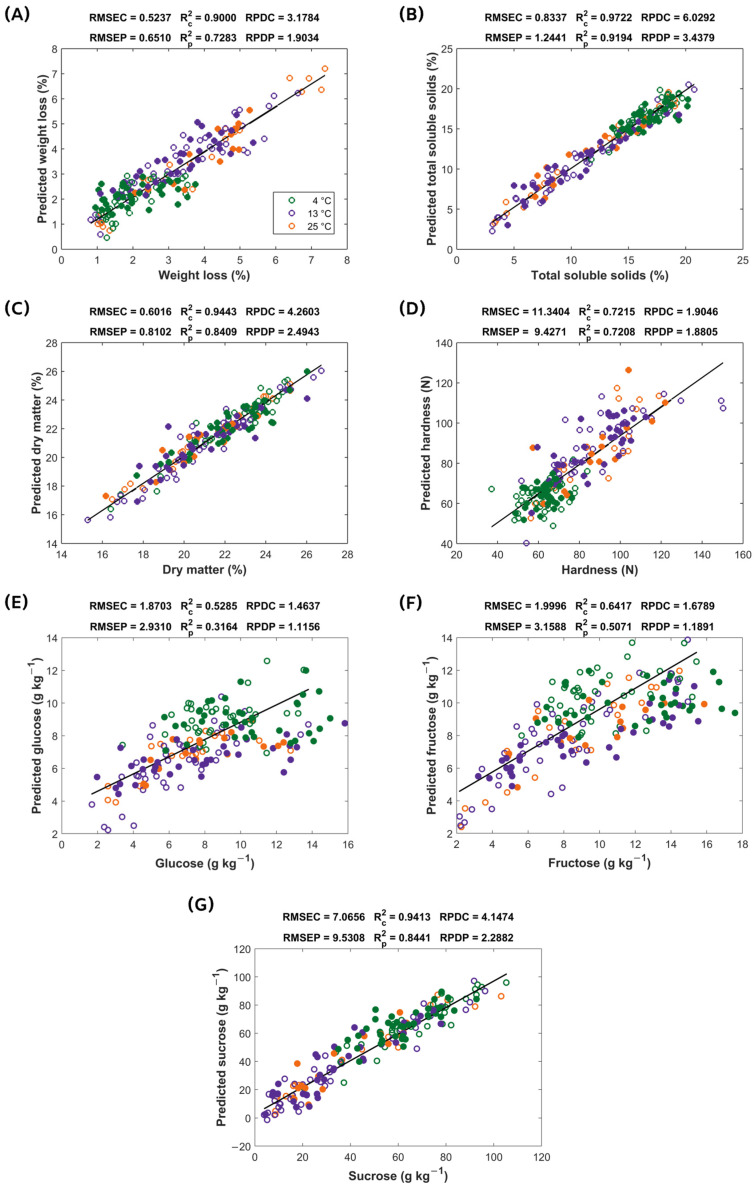
Predicted quality parameters based on the NIR spectral data using PLS (**A**) weight loss, (**B**) total soluble solids, (**C**) dry matter, (**D**) hardness, (**E**) glucose, (**F**) fructose, and (**G**) sucrose. The open and closed symbols represent the training and test samples, respectively. RMSEC = root mean squared error of calibration, RMSEC = root mean squared error of prediction, $R_{c}^{2}$ = coefficient of determination for calibration, $R_{p}^{2}$ = coefficient of determination for prediction, RPDC = ratio of performance to deviation for calibration, and RPDP = ratio of performance to deviation for prediction.

To investigate the relationship between the NIR spectral data and corn quality parameters, PLS models were established using the NIR spectra as predictive variables. The prediction results are illustrated in Figure 4, which presents the correlation graphs for predicting weight loss, TSS, DM, hardness, and the sucrose, fructose, and glucose contents of the corn of the training and testing datasets. The developed PLS models yielded R^2^ ranging from 0.53 to 0.97 for the training samples. The values predicted from the NIR spectra of the test samples closely aligned with those of the training set. These findings are consistent with previous studies [11,12,13,14] and suggest that NIR spectroscopy is a viable approach for predicting quality changes in sweet corn during storage.

In this study, a global PLS model was developed using samples from all storage temperatures to capture the overall variability associated with different storage conditions. This approach reflects practical scenarios in which storage temperatures are not fixed and enhances the robustness and generalizability of the model. Although the corn samples were stored at different temperatures, all samples were equilibrated to 25 °C prior to NIR spectral acquisition, ensuring consistent measurement conditions and minimizing temperature-induced spectral variability. It is also noted that samples stored at 4 °C exhibited relatively small physicochemical changes during the experimental period, resulting in a limited dynamic range and consequently lower predictive performance when modeled separately. For comparison, individual PLS models were also constructed for each storage temperature, and the results are provided in Figures S1–S3. These findings support the use of a global model for more reliable prediction across varying storage conditions.

#### 2.3.3. Multivariate Curve Resolution–Alternating Least Squares

**Figure 5:**
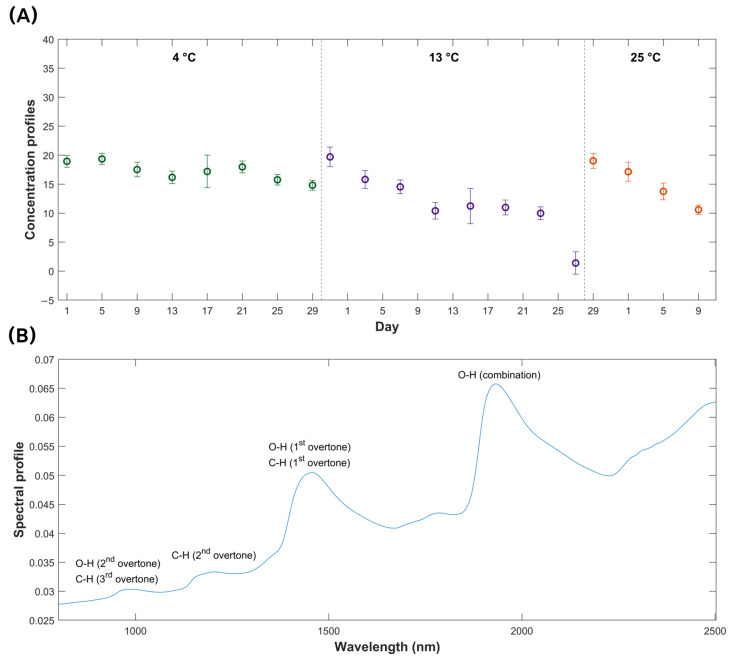
MCR-ALS concentration profiles of component 1 (**A**) and the corresponding spectral profiles (**B**) obtained from the NIR spectra of stored corn samples.

An MCR-ALS model was established using the NIR spectra of the training corn samples. In this study, the model was constructed using the first two components and converged to a relative lack-of-fit (LoF) of 0.75%. The concentration and spectral profiles recovered from MCR-ALS component 1 are presented in Figure 5. As shown in Figure 5A, the concentration scores of the MCR-ALS component exhibited a decreasing trend with increasing storage time under all temperature conditions. The decline was more pronounced at 13 °C and 25 °C than at 4 °C, indicating faster compositional changes at higher temperatures. For example, the concentration values of corn stored at 4 °C decreased slightly from 25.10 ± 3.02 on day 1 to 23.30 ± 1.89 on day 29, whereas those stored at 13 °C showed a more pronounced reduction from 28.29 ± 2.41 to 2.07 ± 2.83 over the same period.

**Figure 6:**
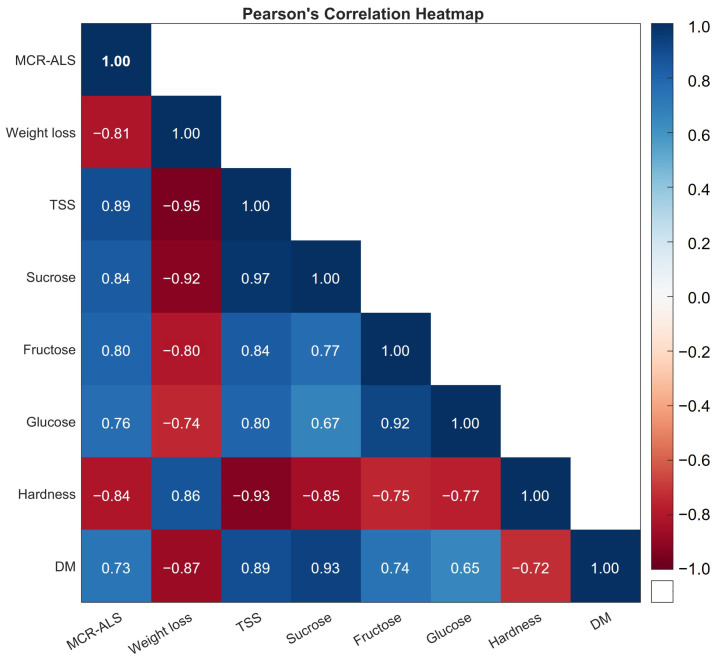
Pearson’s correlations between the MCR-ALS component and the quality parameters of the corn samples.

Figure 5B presents the reconstructed NIR spectral profile obtained from the MCR-ALS model. The reconstructed spectrum exhibits prominent absorption bands that are consistent with the characteristic features observed in the original NIR spectra shown in Figure 3A. Pearson’s correlation coefficients between the concentration scores of MCR-ALS component 1 and the corn quality-related parameters are presented in Figure 6. TSS showed a strong positive relationship with sucrose (0.97), fructose (0.84), and glucose (0.80), as these sugars are the principal contributors to the soluble solids fraction in sweet corn and dominate the refractometric measurement.

The MCR-ALS component also exhibited strong correlations with the measured quality parameters, with coefficients ranging from 0.73 to 0.89. Notably, the component was positively associated with DM, TSS, and all sugar concentrations, whereas weight loss and hardness showed inverse relationships. These findings suggest that the concentration scores obtained from this MCR-ALS analysis effectively capture the major spectral variations related to sugars and moisture and serve as reliable indicators of key quality attributes in sweet corn during storage.

The strong correlations between the MCR-ALS concentration profile and TSS, sucrose, glucose, and fructose indicate that the resolved component primarily reflects changes in the major soluble constituents of sweet corn during storage. Since NIR spectroscopy is sensitive to overtone and combination vibrations of O–H and C–H bonds, changes in soluble sugars are readily captured in the NIR spectra, resulting in strong correlations with the MCR-ALS-derived concentration profile. In contrast, hardness exhibited a weaker correlation because texture is influenced not only by chemical composition but also by structural changes in the kernels, cell wall integrity, moisture redistribution, and tissue turgor during storage. Consequently, hardness cannot be explained solely by the spectral changes associated with soluble chemical constituents.

### 2.4. Multivariate Shelf-Life Prediction Using NIR Spectra

#### 2.4.1. Shelf-Life Prediction Based on MCR-ALS

Table 4 summarizes the kinetic parameters estimated from the zero-order model of the MCR-ALS component at each storage temperature. The corresponding results for the first- and second-order models are provided in Table S2. Using the zero-order kinetics, the variation in the MCR-ALS scores was described with R^2^ values of 0.6860, 0.8292, and 0.9864 at 4, 13, and 25 °C, respectively. The rate constants (*k*) increased with temperature, reaching 0.1322, 0.4976, and 0.7182 day^−1^ at 4, 13, and 25 °C, respectively, indicating faster degradation under higher-temperature conditions. Applying the same cut-off criterion, the predicted shelf-life values were 41.3, 11.0, and 8.9 days at 4, 13, and 25 °C, respectively.

**Figure 7:**
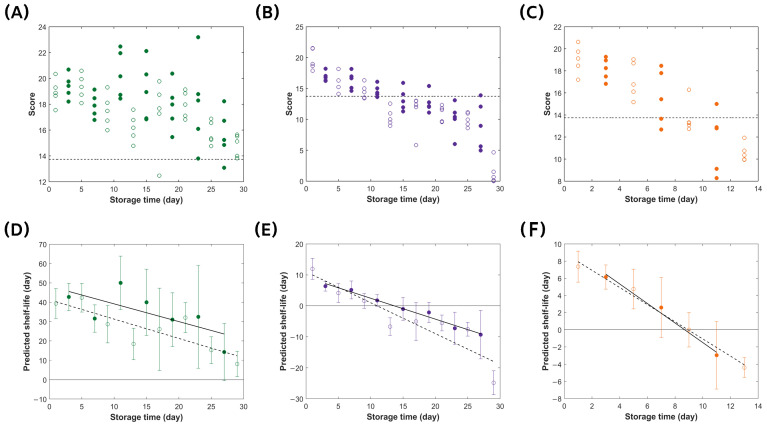
MCR-ALS concentration scores of the test corn during storage using the zero-order kinetic model of (**A**) 4 °C, (**B**) 13 °C and (**C**) 25 °C. The dashed lines represent the cut-off criterion. The open and closed symbols represent the training and test samples, respectively. Predicted shelf life of the training and test corn samples stored at (**D**) 4 °C, (**E**) 13 °C and (**F**) 25 °C. Values are presented mean ± standard deviation.

To demonstrate the application of NIR spectroscopy for shelf-life estimation, the developed NIR–MCR-ALS model was applied to the test corn samples. The MCR-ALS concentration scores were predicted from the recorded NIR spectra and subsequently used to estimate the shelf life of the test samples. Based on the zero-order kinetics of the MCR-ALS component, the relationship between storage time and predicted shelf life is illustrated in Figure 7. Training and test samples are represented by open- and closed-circle symbols, respectively, and the predicted shelf life are presented as mean values with standard deviations. The cut-off value at each storage temperature is indicated by a horizontal black line.

As shown in Figure 7B,C, corn stored at 13 and 25 °C exhibited more rapid deterioration than that stored at 4 °C. At 25 °C, the quality indicator of the test samples began to fall below the cut-off value on day 7, and most samples exhibited predicted shelf-life values below zero by day 13. In Figure 7D–F, the point at which the regression line crosses SL = 0 (the horizontal solid line) represents the predicted end of shelf life. At 25 °C, the estimated shelf life was 8.9 days for the training set and 8.7 days for the validation set. The close agreement between these values demonstrates good model consistency and indicates that sweet corn stored at 25 °C reaches the end of its acceptable quality after approximately 9 days.

At 13 °C, the predicted end of shelf life differed between the training and validation sets, with SL = 0 occurring at 11.0 and 13.6 days, respectively. In Figure 7B, the first signs of deterioration were observed around day 9, and an increasing number of samples were predicted to be expired after day 13. All samples fell below the cut-off criterion by day 21. These findings are consistent with the external appearance shown in Figure 1, where visible quality degradation became apparent around day 20. The results suggest that the NIR–MCR-ALS approach was able to detect chemical changes associated with deterioration earlier than visual observations, particularly at the lower storage temperature.

#### 2.4.2. MASLT Model Developed Using NIR Spectral Data

Table 5 lists the kinetic model constants, including the activation energy (*E_a_*) and acceleration factor (α_T_) the zero-order model. The corresponding results for the first- and second-order models are provided in Table S3. The *E_a_* value was 54.05 kJ mol^−1^, and the Arrhenius model provided a good fit with the R^2^ value of 0.8387, indicating that the degradation of sweet corn followed temperature-dependent kinetics. The acceleration factors were subsequently used to predict shelf life under different storage temperatures.

The shelf-life cut-off was established using the training samples stored at 25 °C for 9 days, at which point the corn remained acceptable for consumption, with green husks and kernels showing no visible signs of mold (Figure 1). This condition corresponded to an MCR-ALS concentration score of 13.74. Based on this criterion, the predicted shelf life at 25 °C was 8.9 days, corresponding to a relative error of 1.1%.

Applying the calculated acceleration factors, corn stored at 13 °C was predicted to have a shelf life of 12.9 days. This prediction was consistent with the observed changes in DM, glucose, and fructose contents, which declined to levels comparable to those of samples stored at 25 °C for 9 days (Table S1). For example, the DM content of corn stored at 13 °C for 13 days was 20.47 ± 1.13%, which was comparable to the value of 19.72 ± 1.96% observed for corn stored at 25 °C for 9 days. Similarly, the glucose content at 13 °C on day 13 (6.69 ± 1.62 g kg^−1^) was comparable to that at 25 °C on day 9 (7.19 ± 2.10 g kg^−1^). Weight loss and hardness exhibited similar trends.

In contrast, corn stored at 4 °C exhibited only minor quality changes throughout the experimental period, and the model predicted a shelf life of 48.5 days under these postharvest conditions. This prediction is in agreement with the findings of Jomnong et al. [17], who reported that sweet corn can be stored for approximately two months at 4 °C. These results support the suitability of the Arrhenius-based NIR–MCR-ALS approach for predicting sweet corn shelf life across different storage temperatures.

#### 2.4.3. Evaluation Using Repeated Non-Destructive Monitoring

**Figure 8:**
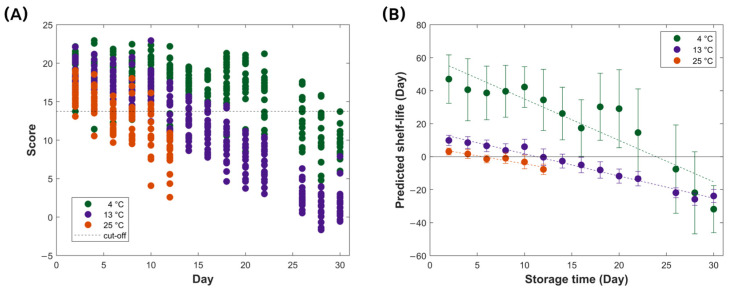
(**A**) Predicted MCR-ALS concentration scores of the additional test samples during storage using the zero-order kinetic model. The dashed line represents the shelf-life cut-off criterion. (**B**) Predicted shelf life of the additional test samples stored at 4, 13, and 25 °C. Values are presented as mean ± standard deviation.

To further investigate the performance of the developed NIR–MCR-ALS framework under repeated non-destructive monitoring, the model was applied to an independent set of sweet corn samples obtained from a separate harvest batch. Unlike the model development dataset, the same corn ears were repeatedly measured throughout storage after husk removal, allowing continuous monitoring of quality deterioration without destructive physicochemical analyses. Figure 8A presents the predicted MCR-ALS concentration scores as a function of storage time based on the zero-order kinetic model, with the shelf-life cut-off value (13.74) indicated by the dashed line. The corresponding predicted shelf lives are shown in Figure 8B.

As expected, the predicted MCR-ALS concentration scores indicated more rapid quality deterioration for samples stored at 25 and 13 °C than for those stored at 4 °C. At 25 °C, the predicted concentration scores of many samples decreased below the cut-off value by Day 6, whereas the corresponding transition occurred on Day 11 for samples stored at 13 °C. Thereafter, all samples stored at 25 and 13 °C exhibited predicted shelf-life values below zero by Days 12 and 22, respectively, indicating complete loss of acceptable quality under these storage conditions.

For samples stored at 4 °C, deterioration occurred earlier than predicted by the kinetic model developed using the model development dataset, with most samples falling below the cut-off criterion within 30 days. This difference is likely attributable to the modified experimental protocol. Unlike the model development dataset, the husks were removed before repeated NIR measurements, and the same corn ears were repeatedly exposed to ambient conditions during spectral acquisition. These factors may have accelerated moisture loss and microbial activity, resulting in a shorter observed shelf life.

Overall, the predicted deterioration profiles remained consistent with the expected temperature-dependent degradation behavior observed in the model development dataset. Although this repeated-monitoring experiment was not intended as a formal external validation because destructive physicochemical analyses were not performed, the results demonstrate that the proposed NIR–MCR-ALS framework can consistently monitor the progression of quality deterioration under repeated non-destructive measurements and modified sample handling conditions.

### 2.5. Limitations and Future Perspectives

Although the proposed NIR–MCR-ALS framework demonstrated promising performance under the experimental conditions investigated in this study, the model was developed using a single sweet corn cultivar (Pure White Hokkaido) collected from one production region during a single harvest season. Consequently, the current model should be regarded as cultivar- and harvest-specific, and its transferability to other cultivars, harvest seasons, and production regions has not yet been established. Variations in genetic background, environmental conditions, cultivation practices, and postharvest physiology may influence both the NIR spectral characteristics and the degradation behavior of sweet corn, potentially affecting model performance. Therefore, future studies should evaluate the proposed framework using independent datasets representing different cultivars, harvest seasons, and production regions. If systematic spectral differences are observed, model updating or calibration transfer strategies may provide an effective means of extending the applicability of the proposed framework.

In addition, the present study focused on objective physicochemical quality parameters as indicators of quality deterioration. Although these parameters successfully described the degradation process, sensory quality, including taste, aroma, texture, and consumer perception, was not evaluated. Future studies should integrate sensory evaluation with the proposed NIR–MCR-ALS framework to further establish the relationship between the predicted quality indices and consumer acceptance.

## 3. Materials and Experimental Methods

### 3.1. Chemicals

Analytical standards, including glucose, fructose, and sucrose, were obtained from Sigma-Aldrich Chemical Co. (St. Louis, MO, USA). Deionized water and ethanol were purchased from RCI Labscan Ltd. (Bangkok, Thailand) and were of analytical grade. Water and acetonitrile used for chromatographic analysis were also purchased from RCI Labscan Ltd. (Bangkok, Thailand) and were of HPLC grade.

### 3.2. Corn Samples and NIR Spectral Acquisition

#### 3.2.1. Corn Samples for Model Development and Evaluation

Pure White Hokkaido sweet corn was harvested at commercial maturity (18–22 days after pollination) in November 2024 from an agricultural farm in San Sai District, Chiang Mai Province, Thailand. Only ears of uniform size and free from visible pest or disease symptoms were selected. After harvest, the corn was transported to the laboratory within one hour for sample preparation and storage. Prior to storage, the dirty outer husks were removed, while the inner husks remained intact. Each corn ear was individually packed in a polyethylene bag perforated with two holes on opposite sides to facilitate ventilation.

The corn samples were randomly assigned to storage at 4, 13, or 25 °C in temperature-controlled incubators, representing refrigerated storage, intermediate retail storage conditions, and ambient tropical storage, respectively [28,29]. To minimize moisture loss, a tray containing water was placed inside each incubator, and the water level was maintained throughout the storage period.

Near-infrared (NIR) spectra were acquired in reflectance mode using an FT-NIR spectrometer (MPA, Bruker Optik GmbH, Ettlingen, Germany) equipped with a fiber-optic probe over the spectral range of 800–2500 nm at 25 °C. For each corn ear, ten spectra were collected from the central region of the cob and averaged prior to subsequent chemometric analysis.

For model development, corn samples stored for Days 1, 5, 9, …, 29 were assigned to the training set. On each designated storage day, the husk was removed, NIR spectra were acquired, and the samples were immediately subjected to destructive reference analyses, including total soluble solids (TSS), dry matter (DM), weight loss, hardness, sucrose, glucose, and fructose contents. A total of 120 corn samples (3 storage temperatures × 8 storage days × 5 biological replicates) were used to establish the kinetic degradation models.

An independent test set consisting of corn samples stored for Days 3, 7, 11, …, 27 was reserved for model evaluation. These samples were processed using the same experimental protocol as the training set, including a single NIR measurement followed immediately by destructive physicochemical analyses. A total of 105 corn samples (3 storage temperatures × 7 storage days × 5 biological replicates) were used for independent evaluation of the developed NIR prediction models.

#### 3.2.2. Additional Test Samples for Repeated Non-Destructive Monitoring

To further examine the practical applicability of the proposed NIR–MCR-ALS framework, an additional experiment was conducted using a separate harvest batch of Pure White Hokkaido sweet corn. Unlike the model development dataset described above, the same corn ears were repeatedly monitored throughout storage using NIR spectroscopy without destructive physicochemical analyses.

The corn samples were stored under the same three temperature conditions (4, 13, and 25 °C) using identical storage procedures. On Day 2, the husks were removed to enable direct NIR measurement. Following spectral acquisition, each corn ear was repacked in a new perforated polyethylene bag and returned to its original storage condition. NIR spectra were subsequently collected from the same corn ears every two days (Days 2, 4, 6, …, 30), allowing continuous monitoring of quality changes throughout storage.

For each measurement, ten spectra were collected from the central region of each corn ear and averaged before analysis. The repeated monitoring dataset consisted of 20 biological replicates at each storage temperature, resulting in a total of 680 NIR spectra: 560 spectra from samples stored at 4 and 13 °C (2 storage temperatures × 14 storage days × 20 biological replicates) and 120 spectra from samples stored at 25 °C (1 storage temperature × 6 storage days × 20 biological replicates).

Because the same corn ears were repeatedly measured after husk removal and no destructive reference analyses were performed, this dataset was not intended as a formal external validation set. Instead, it was used to examine whether the developed NIR–MCR-ALS framework could consistently track the progression of quality deterioration under repeated non-destructive measurements and modified sample handling conditions.

### 3.3. Physiochemical Analysis

Quality-related parameters, including weight loss, DM, hardness, TSS, and concentrations of fructose, glucose, and sucrose, were quantified during storage. The procedures for determining corn quality parameters followed the method described by Jomnong et al. [17].

Briefly, weight loss was measured according to AOAC [30] using an electronic balance (MonoBloc, Mettler Toledo, Switzerland) and calculated as the percentage difference between the sample weight before storage and that on the analysis day. The DM value was obtained from kernels dried at 70 °C for 72 h in a hot-air oven (Venticell 111, MMM Medcenter Einrichtungen GmbH, Munich, Germany) and expressed as the mean of three replicates. Hardness was measured using a texture analyzer (TA.Xt plus, Texture Technologies Corp., Godalming, UK) with a 50 mm cylindrical probe at 1 mm/s. Five kernels per ear were analyzed, and the mean value was reported. The TSS value was obtained from 2 mL of kernel juice using a digital refractometer (PAL-1, Atago Ltd. Co., Tokyo, Japan), with the mean of three measurements recorded for each ear.

For sugar analysis (fructose, glucose, and sucrose), corn kernels from the middle of each ear were excised, finely chopped, and a 5.00 g portion was placed in a 50 mL centrifuge tube. Ten milliliters of 80:20 ethanol–water solution [31] was added, and the mixture was vortexed for 1 min (G560E Genie 2, Scientific Industries Inc., Bohemia, NY, USA), heated at 80 °C for 10 min (WNB 14, Memmert GmbH Co., Schwabach, Germany), sonicated for 10 min (TRU-SWEEP model-175T, Crest Ultrasonics Corp., Trenton, NJ, USA), and centrifuged at 5000 rpm for 15 min (Universal 320R, Hettich GmbH, Tuttlingen, Germany). The extraction was repeated twice, and the combined extract was adjusted to 25 mL with the same solvent. The solution was then centrifuged at 15,000 rpm for 15 min (Mikro 22R, Hettich GmbH, Tuttlingen, Germany), and the supernatant was filtered (0.45 µm nylon filter, Agilent Technologies Inc., Santa Clara, CA, USA) prior to HPLC analysis using a KBP 6395 FL system (Agilent Technologies Inc., Santa Clara, CA, USA) equipped with a Purospher NH_2_ column (5 µm, 150 × 4.6 mm; Agilent Technologies Inc., Santa Clara, CA, USA) at 40 °C. The mobile phase consisted of 80:20 acetonitrile–water at a flow rate of 1 mL/min with refractive index detection.

### 3.4. Chemometric Modellings

#### 3.4.1. Spectral Resolution Using Multivariate Curve Resolution–Alternating Least Squares

MCR-ALS was applied to resolve a bilinear structure of the NIR spectral data, which resembles the generalization of the Lambert–Beer law [32]. The MCR-ALS resolution yielded both concentration and spectral profiles. The concentration profile can be related to the variation in NIR spectra as a function of storage time, which, in this context, can be further used to establish kinetic degradation models.

The MCR-ALS bilinear model can be expressed as:

***X*** = ***CS***′+ ***E*** (1)

where ***X*** is a data matrix of the NIR spectral data, ***C*** and ***S*** represent the concentration and spectral profiles, respectively, and ***E*** is the residual matrix containing unexplained variations not captured by the bilinear model. ***X*** is expressed as the dot product of ***C*** and ***S′***, described using *A* components. The optimal number of *A* components was determined based on singular value decomposition (SVD).

Several data preprocessing techniques, such as square-root scaling, standard normal variate (SNV), multiplicative scatter correction (MSC), and Savitzky–Golay filtering, were evaluated. Among these, square-root scaling produced the most satisfactory results. Therefore, the NIR spectra were preprocessed using square-root scaling prior to MCR-ALS, PCA, and PLS analyses.

In this study, non-negativity constraints were applied during iteration [33] to ensure that the concentration and spectral profiles remained non-negative, consistent with the Lambert–Beer law.

#### 3.4.2. Principal Component Analysis

PCA, a widely used exploratory tool in chemometrics [34], was performed to reveal a characteristic pattern in the NIR spectra. PCA produces a set of new variables, referred to as principal components (PCs), which facilitate visualization of the variability in the dataset.

The PCA-based mathematical transformation of the data matrix ***X*** can be expressed as:

***X*** = ***TP***′ + ***E*** (2)

where ***T***, ***P***, and ***E*** denote score, loading, and variance residual matrices, respectively. In most cases, the product matrix ***TP***′, constructed from the first few PCs, captures the majority of the systematic variation, thereby illustrating the multivariate patterns present in the data.

#### 3.4.3. Partial Least Squares Regression

PLS regression [35] was applied to NIR spectral data to predict quality parameters. As a standard and effective regression analysis for NIR spectral data, PLS maximizes the covariance between predictor and response variables to construct robust calibration models [36]. Model performance was assessed using the root mean square error (RMSE):

(3) $$RMSE=\;\sqrt{\frac{\sum_{i=1}^{N}{\left({\hat{c}}_{i}-c_{i}\right)}^{2}}{N-1}}$$

where ${\hat{c}}_{i}$ is the predicted value, $c_{i}$ is the reference value, and *N* is the number of samples. The coefficient of determination for calibration ($R_{c}^{2}$) [37] was also calculated as:

(4) $$R_{c}^{2}=1-\frac{\sum_{i=1}^{N}{\left({\hat{c}}_{i}-c_{i}\right)}^{2}}{\sum_{i=1}^{N}{\left(c_{i}-\bar{c}\right)}^{2}}$$

where $\bar{c}$ is the mean of the reference values. The value of $R_{c}^{2}$ approaching 1.0 indicates excellent calibration performance. The optimal number of latent variables for the PLS prediction was determined by leave-one-out cross-validation [38].

### 3.5. Shelf-Life Prediction Models

The MASLT approach was applied using the concentration profile that represents the degradation trend observed in the NIR spectra. The temporal change in this component was plotted as a function of storage time for each temperature condition, and the resulting degradation patterns were modeled to determine the reaction kinetics and shelf-life parameters.

#### 3.5.1. Kinetic Analysis

The kinetic order was evaluated by comparing the coefficients of determination ($R^{2}$) obtained from the following equations:

(5) $$Zero-order\;:A=A_{0}-k_{0}t$$

(6) $$First-order\;:ln\;A={ln\;A}_{0}-k_{1}t$$

(7) $$Second-order\;:\frac{1}{A}=\frac{1}{A_{0}}+k_{2}t$$

where $A_{0}$ and A denote the MCR-ALS concentration scores or quality parameter values at the initial time and at a specific storage time, respectively. The rate constants obtained from the zero-order ($k_{0}$), first-order ($k_{1}$), and second-order ($k_{2}$) kinetic models were then used to develop shelf-life prediction models for each storage temperature as follows:

(8) $${SL}_{0}=\frac{A_{0}-A_{C}}{k_{0}}$$

(9) $${SL}_{1}=\frac{\ln A_{0}-\ln A_{C}\;}{k_{1}}$$

(10) $${SL}_{2}=\frac{\frac{1}{A_{0}}-\frac{1}{A_{C}}}{k_{2}}$$

where ${SL}_{0}$, ${SL}_{1}$ and ${SL}_{2}$ represent the shelf-life (days) estimated using the degradation rate constants obtained from the zero-order ($k_{0}$), first-order ($k_{1}$), and second-order ($k_{2}$) kinetic models, respectively. $A_{C}$ represents the cut-off value of the MCR-ALS concentration or quality parameter.

To demonstrate the proposed approach, corn samples stored at 25 °C for 9 days were selected to define the shelf-life cut-off criterion, as their quality remained acceptable, with green husks and no visible mold growth or kernel deterioration. Therefore, the average quality parameter value of these samples was used as the cut-off criterion in the kinetic degradation models.

#### 3.5.2. Arrhenius Model

The reaction order at each storage temperature was evaluated using the zero-, first-, and second-order kinetic models, as well as the Arrhenius relationship describing the temperature dependence of the rate constant (*k*). Accordingly, the Arrhenius equation was applied as follows:

(11) $$\ln k=\ln k_{ref}-\frac{E_{a}}{R}(\frac{1}{T}-\frac{1}{T_{ref}})$$

where $k_{ref}$ denotes reaction rate constant at reference temperature, $E_{a}$ presents activation energy (J mol^−1^), R is the ideal gas constant (8.314 J mol^−1^K^−1^), and T and $T_{ref}$ are absolute and reference temperature (K), respectively [39].

After obtaining the rate constants at different temperatures, an acceleration factor ($\alpha_{T+\delta T,T}$) was calculated to estimate the relationship between degradation rates under accelerated and reference conditions:

(12) $$\alpha_{T+\delta T,T}=\frac{k_{T+\delta T}}{k_{T}}$$

where $k_{T+\delta T}\;$ and $k_{T}$ are the rate constants at the accelerated and reference temperatures, respectively.

The shelf life at a given storage temperature was then predicted from the critical degradation time obtained under accelerated conditions according to:

(13) $${SL}_{T}=\alpha_{T+\delta T,T}\times {time}_{crit,T+\delta T}$$

where ${SL}_{T}$ represents the estimated shelf life (days) at storage temperature T, derived from samples stored at an accelerated temperature of T+δT. In this research, the NIR spectral data analysis, PCA and PLS were carried out using MATLAB R2019b (The MathWorks Inc., Natick, MA, USA). The MCR-ALS analysis was carried out using the MCR-ALS 2.0 toolbox available at http://www.mcrals.info/ (accessed on 1 June 2025).

## 4. Conclusions

This study demonstrated that NIR spectroscopy combined with MCR-ALS can effectively predict the shelf life of sweet corn in a rapid and non-destructive manner. The MCR-ALS approach successfully decomposed the NIR spectra into chemically interpretable profiles, revealing compositional and structural changes during storage. Kinetic modeling of the MCR-ALS concentration profiles using zero-order and Arrhenius models yielded temperature-dependent degradation rates and accurate shelf-life predictions consistent with experimental observations. Compared with conventional univariate models, the proposed NIR–MCR-ALS framework provides an integrated, interpretable, and efficient approach for assessing postharvest quality. The method shows strong potential for rapid, non-destructive shelf-life monitoring and could be readily integrated into routine postharvest quality assessment of sweet corn.

Beyond its analytical performance, the proposed NIR–MCR-ALS framework offers several practical advantages for postharvest quality management. Because NIR spectra were acquired directly from intact sweet corn cobs without damaging the samples, the framework enables rapid and repeated quality assessment throughout storage, providing a significant advantage over conventional destructive physicochemical analyses. This capability has considerable potential to support postharvest supply chain management by facilitating timely decision-making during storage, transportation, and distribution, thereby helping to optimize cold chain logistics, reduce postharvest losses and food waste, and ensure that products reach consumers with acceptable quality. Future work should further evaluate the proposed framework using different sweet corn cultivars, harvest seasons, production regions, and other agricultural commodities, as well as under diverse commercial postharvest scenarios, including cold storage, ambient transportation, and modified-atmosphere packaging, to further demonstrate its robustness and broader practical applicability.

## Figures and Tables

**Table 1:** Comparison of the chemometric methods used in this study.

Method	Primary Objective	Output	Advantages	Role in this Study
PCA	Exploratory analysis	Principal component scores and loadings	Reveals clustering and major spectral variation	Explore temperature- and storage-related spectral changes
PLS	Regression	Predicted physicochemical parameters	Quantitative prediction of individual quality attributes	Predict individual physicochemical quality parameters
MCR-ALS	Spectral resolution	Concentration profiles and pure spectral estimates	Resolves dominant degradation-related chemical changes and provides interpretable latent variables	Derive an integrated quality indicator for kinetic degradation modeling and shelf-life prediction

**Table 2:** Postharvest quality parameters of corn during storage at different temperatures.

Quality Parameter	4 °C		13 °C		25 °C	
	Range	Mean ± SD	Range	Mean ± SD	Range	Mean ± SD
Weight loss (%)	0.95–3.75	2.01 ± 0.77 ^c^	0.82–6.62	3.32 ± 1.36 ^b^	1.02–7.38	3.98 ± 1.79 ^a^
TSS (%)	11.75–20.20	16.76 ± 1.89 ^a^	3.10–20.75	11.09 ± 4.59 ^b^	3.30–19.05	11.14 ± 4.82 ^b^
DM (%)	16.41–26.02	22.34 ± 2.00 ^a^	15.28–26.72	21.00 ± 2.38 ^b^	16.17–25.19	20.95 ± 2.41 ^b^
Hardness (N)	37.03–83.85	62.71 ± 8.20 ^b^	51.65–150.25	87.47 ± 19.47 ^a^	56.26–121.95	87.91 ± 18.72 ^a^
Glucose (g kg^−1^)	3.40–15.00	9.51 ± 2.52 ^a^	1.68–15.82	7.02 ± 3.14 ^b^	2.58–12.79	7.33 ± 2.72 ^b^
Fructose (g kg^−1^)	5.64–17.58	10.94 ± 2.97 ^a^	2.17–15.52	8.36 ± 3.77 ^b^	2.26–15.86	8.94 ± 3.49 ^b^
Sucrose (g kg^−1^)	20.74–105.22	64.3 ± 16.81 ^a^	3.88–96.35	35.42 ± 25.09 ^b^	8.58–103.09	40.26 ± 26.66 ^b^

Values are expressed as mean ± standard deviation (SD). Different letters within the same row indicate significant differences (Tukey’s test, *p* < 0.05).

**Table 3:** Estimated kinetic parameters of quality attributes obtained from zero-, first-, and second-order reaction models.

Quality Parameter	Cut-Off	T	Zero Order					First Order					Second Order				
		(°C)	*a*	*k*	R^2^	RMSE*	SL (Day)	*a*	*k*	R^2^	RMSE*	SL (Day)	*a*	*k*	**R^2^**	RMSE*	**SL (Day)**
Weight loss (%)	4.76	4	0.9753	−0.0670	0.8404	0.2677	56.4	0.1120	−0.0344	0.9056	0.1018	42.1	0.8436	0.0187	0.9311	0.0467	33.8
		13	1.0377	−0.1563	0.9690	0.2561	23.8	0.2673	−0.0553	0.8912	0.1769	23.4	0.7494	0.0237	0.7208	0.1355	22.7
		25	0.7053	−0.4661	0.9876	0.2338	8.7	0.1913	−0.1430	0.9288	0.1770	9.6	0.7804	0.0565	0.7864	0.1316	10.1
TSS (%)	8.23	4	19.1808	0.1625	0.9202	0.4385	67.4	2.9601	0.0098	0.9209	0.0262	87.3	0.0514	−0.0006	0.9168	0.0016	118.8
		13	18.8267	0.5234	0.9873	0.5443	20.2	3.0956	0.0545	0.9375	0.1289	18.1	0.0164	−0.0068	0.7722	0.0339	15.4
		25	19.6998	1.2018	0.9990	0.1697	9.5	3.1416	0.1224	0.9660	0.1027	8.4	0.0165	−0.0147	0.8666	0.0259	7.1
DM (%)	19.65	4	24.2421	0.1260	0.8835	0.4192	36.4	3.1904	0.0057	0.8808	0.0191	37.5	0.0410	−0.0003	0.8765	0.0009	38.5
		13	23.5789	0.1779	0.7937	0.8313	22.1	3.1625	0.0084	0.7999	0.0385	21.9	0.0422	−0.0004	0.8033	0.0018	21.8
		25	24.1873	0.4499	0.8859	0.7220	10.1	3.1878	0.0209	0.9059	0.0301	10.0	0.0412	−0.0010	0.9247	0.0012	10.0
Hardness (N)	103.1	4	61.3971	−0.0617	0.0128	4.9645	674.9	4.1151	−0.0009	0.0106	0.0817	562.9	0.0164	0.0000	0.0086	0.0014	484.0
		13	60.3294	−1.8327	0.9313	4.5609	23.3	4.1358	−0.0213	0.9308	0.0532	23.5	0.0157	0.0003	0.9023	0.0008	23.5
		25	64.1106	−3.3602	0.9842	1.9035	11.6	4.1810	−0.0395	0.9711	0.0305	11.5	0.0151	0.0005	0.9515	0.0005	11.4
Glucose (g kg^−1^)	6.8	4	7.3577	−0.1011	0.8565	0.3793	5.5	2.0015	−0.0117	0.8428	0.0462	7.3	0.1346	0.0014	0.8249	0.0057	9.2
		13	9.4452	0.1967	0.8034	0.8919	13.5	2.3267	0.0342	0.7614	0.1757	12.0	0.0757	−0.0066	0.6601	0.0437	10.8
		25	9.9199	0.4354	0.8639	0.7728	7.2	2.3896	0.0748	0.7920	0.1714	6.3	0.0701	−0.0139	0.7273	0.0380	5.5
Fructose (g kg^−1^)	8.93	4	10.3508	0.0112	0.0418	0.4916	126.9	2.3348	0.0010	0.0366	0.0482	142.1	0.0970	−0.0001	0.0318	0.0047	160.0
		13	12.6760	0.3209	0.8498	1.2363	11.7	2.6566	0.0460	0.8308	0.1903	10.2	0.0397	−0.0079	0.6906	0.0482	9.2
		25	12.8850	0.6279	0.8708	1.0816	6.3	2.6589	0.0856	0.8149	0.1825	5.5	0.0495	−0.0130	0.7418	0.0344	4.8
Sucrose (g kg^−1^)	16.09	4	91.2879	1.6421	0.9669	2.7830	45.8	4.5491	0.0251	0.9675	0.0422	70.5	0.0099	−0.0004	0.9420	0.0009	131.5
		13	77.9681	2.8017	0.9079	8.1789	22.1	4.6319	0.0915	0.9872	0.0954	20.3	−0.0140	−0.0046	0.8089	0.0203	16.7
		25	89.0132	6.5391	0.9584	6.0893	11.2	4.7474	0.1841	0.9747	0.1327	10.7	−0.0063	−0.0072	0.9047	0.0104	9.6

R^2^ = coefficient of determination; RMSE* = root mean squared error of calibration of the kinetic degradation model.

**Table 4:** Estimated kinetic parameters obtained from the zero-order reaction model using the NIR–MCR-ALS concentration profile.

T (°C)	*A*_0_	*k*	R^2^	SL_train_ (Day)	SL_test_ (Day)
4	19.1983	0.1322	0.6860	41.3	52.7
13	19.2158	0.4976	0.8292	11.0	13.6
25	20.1554	0.7182	0.9864	8.9	8.7

R^2^ = coefficient of determination of the kinetic degradation model; SL_train_ and SL_test_ = predicted shelf life for training and test samples, respectively.

**Table 5:** Activation energy constants (*E*_a_) of the Arrhenius equation and shelf-life predictions from NIR–MCR-ALS concentration profile using the zero-order reactions.

T	*k* (Day^−1^)	*α*_T_ (T,4)	*E*_a_	R^2^	Cut-Off	SL	Relative Error
(°C)			(kJ mol^−1^)			(Day)	(%)
4	0.1322	1.0000	54.05	0.8387	13.74	48.5	-
13	0.4976	3.7637				12.9	-
25	0.7182	5.4330				8.9	1.1

R^2^ = coefficient of determination of the Arrhenius model; *α*_T_ = acceleration factor relative to the reference temperature; SL = predicted shelf life.

## Data Availability

The data presented in this study are available on request from the corresponding author.

## Supplementary Materials

The following supporting information can be downloaded at: https://www.mdpi.com/article/10.3390/molecules31142512/s1, Figure S1. Correlation plots between measured and predicted quality parameters based on NIR spectral data of training and test samples (open- and closed-circle symbols, respectively) stored at 4 °C using PLS. Figure S2. Correlation plots between measured and predicted quality parameters based on NIR spectral data of training and test samples (open- and closed-circle symbols, respectively) stored at 13 °C using PLS. Figure S3. Correlation plots between measured and predicted quality parameters based on NIR spectral data of training and test samples (open- and closed-circle symbols, respectively) stored at 25 °C using PLS. Table S1. Quality attributes of sweet-corn samples stored at 4 °C, 13 °C, and 25 °C over 29 days. Values are expressed as mean ± standard deviation (SD). Different letters within the same row indicate significant differences among storage times at the same temperature (Tukey’s multiple comparison test, *p* < 0.05); Table S2. Estimated kinetic parameters obtained from zero-, first-, and second-order reaction models using the NIR–MCR-ALS concentration profile. Table S3. Activation energy constants (*E_a_*) of the Arrhenius equation and shelf-life predictions from NIR–MCR-ALS concentration profile using the zero, first and second-order reactions.

## References

[1] Mahajan P.V., Caleb O.J., Singh Z., Watkins C.B., Geyer M. (2014). Postharvest treatments of fresh produce. Philos. Trans. A Math. Phys. Eng. Sci..

[2] Gallagher M.J.S., Mahajan P.V., Yan Z., Kilcast D., Subramaniam P. (2011). Modelling chemical and physical deterioration of foods and beverages. Food and Beverage Stability and Shelf Life.

[3] Singh I., Langyan S., Yadava P. (2014). Sweet corn and corn-based sweeteners. Sugar Technol..

[4] Kumar N., Kachhadiya S., Nayi P. (2020). Storage stability and characterization of biochemical, rehydration and colour characteristics of dehydrated sweet corn kernels. J. Stored Prod. Res..

[5] Calligaris S., Lucci P., Milani A., Rovellini P., Lagazio C., Conte L., Nicoli M.C. (2022). Application of accelerated shelf-life test (ASLT) procedure for the estimation of the shelf-life of extra virgin olive oils: A validation study. Food Packag. Shelf Life.

[6] Brezmes J., Llobet E., Vilanova X., Orts J., Saiz G., Correig X. (2001). Correlation between electronic nose signals and fruit quality indicators on shelf-life measurements with pinklady apples. Sens. Actuators B Chem..

[7] Mushtaq A., Jabeen A., Yousouf M., Malik M.A., Mukhtar T., Amin T., Showkat S., Rafiq A. (2025). Post-harvest quality management of sweet corn: Disorders, losses and preservation strategies. Food Nutr..

[8] Li Y., Ding S., Wang Y. (2022). Shelf life predictive model for postharvest shiitake mushrooms. J. Food Eng..

[9] Nordey T., Davrieux F., Léchaudel M. (2019). Predictions of fruit shelf life and quality after ripening: Are quality traits measured at harvest reliable indicators?. Postharvest Biol. Technol..

[10] Nicolaï B.M., Defraeye T., de Ketelaere B., Herremans E., Hertog M.L., Saeys W., Torricelli A., Vandendriessche T., Verboven P. (2014). Nondestructive measurement of fruit and vegetable quality. Annu. Rev. Food Sci. Technol..

[11] Gao F., Xing Y., Li J., Guo L., Sun Y., Shi W., Yuan L. (2025). Prediction of total soluble solids in apricot using adaptive boosting ensemble model combined with NIR and high-frequency UVE-selected variables. Molecules.

[12] Eisenstecken D., Panarese A., Robatscher P., Huck C.W., Zanella A., Oberhuber M. (2015). A near infrared spectroscopy (NIRS) and chemometric approach to improve apple fruit quality management: A case study on the cultivars “Cripps Pink” and “Braeburn”. Molecules.

[13] Wang X., Wang X., Guo Y. (2017). Rapidly simultaneous determination of six effective components in Cistanche tubulosa by near infrared spectroscopy. Molecules.

[14] Yang Q., Yang X., Zhang Q., Wang Y., Song H., Huang F. (2020). Quantifying soluble sugar in super sweet corn using near-infrared spectroscopy combined with chemometrics. Optik.

[15] Chaudhry M.M.A., Amodio M.L., Babellahi F., de Chiara M.L.V., Rubio J.M.A., Colelli G. (2018). Hyperspectral imaging and multivariate accelerated shelf life testing (MASLT) approach for determining shelf life of rocket leaves. J. Food Eng..

[16] Cruz-Tirado J.P., Oliveira M., de Jesus Filho M., Godoy H.T., Amigo J.M., Barbin D.F. (2021). Shelf life estimation and kinetic degradation modeling of chia seeds (*Salvia hispanica*) using principal component analysis based on NIR-hyperspectral imaging. Food Control.

[17] Jomnong P., Funsueb S., Thanavanich C., Theanjumpol P., Muenmanee N., Luengwilai K., Saengrayap R., Arwatchananukul S., Chaiwong S., Mahajan P. (2025). Non-destructive shelf-life prediction models for sweet corn via NIR spectroscopy and integrated postharvest quality parameters. Postharvest Biol. Technol..

[18] de Juan A., Jaumot J., Tauler R. (2014). Multivariate curve resolution (MCR). Solving the mixture analysis problem. Anal. Methods.

[19] Garrido M., Rius F.X., Larrechi M.S. (2008). Multivariate curve resolution–alternating least squares (MCR-ALS) applied to spectroscopic data from monitoring chemical reactions processes. Anal. Bioanal. Chem..

[20] Serrano N., Pérez-Ràfols C., Ariño C., Esteban M., Díaz-Cruz J.M. (2020). MCR-ALS of voltammetric data for the study of environmentally relevant substances. Microchem. J..

[21] Khoobi A., Ghoreishi S.M., Masoum S., Behpour M. (2013). Multivariate curve resolution-alternating least squares assisted by voltammetry for simultaneous determination of betaxolol and atenolol using carbon nanotube paste electrode. Bioelectrochemistry.

[22] Balcázar-Zumaeta C.R., Maicelo J.L., Pajuelo-Muñoz A.J., Torrejón-Valqui L., Muñóz-Astecker L.D., Barrena M., Cayo-Colca I.S., Castro-Alayo E.M. (2025). PCA, PLS, and MCR-ALS applied to the analysis of miscibility and quantification of Cupuassu butter, Passion fruit oil and Sacha Inchi oil blended in Cocoa butter. Appl. Food Res..

[23] Marques A.S., Bedia C., Lima K.M.G., Tauler R. (2016). Assessment of the effects of As(III) treatment on cyanobacteria lipidomic profiles by LC-MS and MCR-ALS. Anal. Bioanal. Chem..

[24] Grassi S., Alamprese C., Bono V., Casiraghi E., Amigo J.M. (2014). Modelling milk lactic acid fermentation using multivariate curve resolution-alternating least squares (MCR-ALS). Food Bioprocess Technol..

[25] Le Dréau Y., Dupuy N., Artaud J., Ollivier D., Kister J. (2009). Infrared study of aging of edible oils by oxidative spectroscopic index and MCR-ALS chemometric method. Talanta.

[26] Ubhi G.S., Sadaka S. (2015). Temporal valuation of corn respiration rates using pressure sensors. J. Stored Prod. Res..

[27] Rambla F.J., Garrigues S., de la Guardia M. (1997). PLS-NIR determination of total sugar, glucose, fructose and sucrose in aqueous solutions of fruit juices. Anal. Chim. Acta.

[28] Hong H.T., Phan A.D.T., O’Hare T.J. (2021). Temperature and maturity stages affect anthocyanin development and phenolic and sugar content of purple-pericarp supersweet sweetcorn during storage. J. Agric. Food Chem..

[29] Evans J.A., Scarcelli S., Swain M.V.L. (2007). Temperature and energy performance of refrigerated retail display and commercial catering cabinets under test conditions. Int. J. Refrig..

[30] AOAC (2000). Official Methods of Analysis.

[31] Zhu S., Mount J.R., Collins J.L. (1992). Sugar and soluble solids changes in refrigerated sweet corn (*Zea mays* L). J. Food Sci..

[32] Kumar K., Mishra A.K. (2012). Application of ‘multivariate curve resolution alternating least square (MCR-ALS)’ analysis to extract pure component synchronous fluorescence spectra at various wavelength offsets from total synchronous fluorescence spectroscopy (TSFS) data set of dilute aqueous solutions of fluorophores. Chemom. Intell. Lab. Syst..

[33] Jaumot J., de Juan A., Tauler R. (2015). MCR-ALS GUI 2.0: New features and applications. Chemom. Intell. Lab. Syst..

[34] Brereton R.G. (2003). Chemometrics: Data Analysis for the Laboratory and Chemical Plant.

[35] Geladi P., Kowalski B.R. (1986). Partial least-squares regression: A tutorial. Anal. Chim. Acta.

[36] Funsueb S., Krongchai C., Mahatheeranont S., Kittiwachana S. (2016). Prediction of 2- acetyl-1-pyrroline content in grains of Thai jasmine rice based on planting condition, plant growth and yield component data using chemometrics. Chemom. Intell. Lab. Syst..

[37] Consonni V., Ballabio D., Todeschini R. (2009). Comments on the definition of the Q^2^ parameter for QSAR validation. J. Chem. Inf. Model..

[38] Brereton R.G. (2009). Chemometrics for Pattern Recognition.

[39] van Boekel M.A.J.S. (2008). Kinetic Modeling of Reactions in Foods.

