# Supplementary material for: Comprehensive and Non-Destructive Sweet Corn Shelf-Life Prediction Using Near-Infrared (NIR) Spectroscopy Coupled with Multivariate Curve Resolution-Alternating Least Squares (MCR-ALS) Spectral Resolution
Source: Molecules. 2026 Jul 18;31(14):2512. doi: 10.3390/molecules31142512 (PMC13416319; doi:10.3390/molecules31142512)
Supplement: Supplementary file 1 [file molecules-31-02512-s001.zip › molecules-4399668-supplementary.pdf]

Supplementary materials

Table S1. Quality attributes of sweet-corn samples stored at 4 °C, 13 °C, and 25 °C over 29 days. Values are expressed as mean ± standard deviation (SD). Different letters within the same row indicate significant differences among storage times at the same temperature (Tukey's multiple comparison test, $p < 0.05$).

Quality attributes	T (°C)	Storage time (day)							
		1	3	5	7	9	11	13	15
Weight loss (%)	4	1.22±0.19f	1.11±0.12f	1.25±0.13ef	1.41±0.33def	1.51±0.23cdef	1.57±0.28cdef	1.61±0.17cdef	1.98±0.32bcde
	13	0.99±0.12h	1.56±0.32gh	1.87±0.21fgh	22.22±0.41fg	2.33±0.34efg	2.81±0.40def	3.57±0.64bcd	3.37±0.30cde
	25	1.15±0.12d	2.66±0.59c	3.25±0.69c	4.39±0.49b	4.52±0.55b	4.98±0.17b	6.94±0.40a	
Total soluble solids (%)	4	19.38±0.20a	19.08±0.25ab	17.81±0.71abc	19.02±1.06ab	17.40±1.00abcd	16.73±0.79bcde	17.85±1.08abc	16.78±1.56abcde
	13	18.40±2.00a	17.65±1.18ab	16.96±1.44ab	15.08±0.63bc	13.58±1.28cd	12.77±1.31cd	11.47±0.83de	11.03±0.98def
	25	18.54±0.56a	15.15±1.50b	13.77±0.87b	10.24±2.49c	8.60±1.98c	7.46±0.65c	4.24±1.02d	
Dry matter (%)	4	24.36±0.67a	23.70±0.58ab	23.17±0.94ab	24.35±1.23a	22.72±1.39ab	22.27±1.38ab	23.23±1.90ab	22.02±1.97ab
	13	23.52±2.83a	23.75±1.66a	23.96±1.47a	21.84±1.44ab	21.34±1.71ab	21.65±2.01ab	20.47±1.13ab	21.25±1.53ab
	25	24.52±0.63a	22.12±1.65ab	20.96±2.64b	19.28±2.22b	19.72±1.96b	21.14±0.98ab	18.93±1.21b	
Hardness (N)	4	60.05±9.48ab	69.85±3.56a	66.98±6.37ab	65.35±2.27ab	63.80±5.28ab	59.21±5.87ab	57.78±6.43ab	54.51±4.29ab
	13	59.27±6.01d	68.23±1.87cd	74.74±4.46bcd	75.17±11.37bcd	78.96±8.49bcd	77.33±16.90bcd	80.74±4.78bcd	90.93±10.19abc
	25	66.44±5.56d	73.38±8.07cd	87.09±20.97bcd	84.35±15.72abcd	97.40±9.42abc	107.12±12.02a	105.76±8.54ab	
Glucose ($g\ kg^{-1}$)	4	7.16±2.35a	11.43±2.21a	7.54±1.97a	11.91±3.43a	8.99±2.41a	11.41±1.91a	9.14±1.84a	9.15±2.39a
	13	9.83±2.23abc	10.33±2.27ab	6.61±1.59bcde	12.79±2.05a	8.51±3.58abcd	8.29±2.72bcd	6.69±1.62bcd	8.35±0.91bcd
	25	9.00±2.94ab	10.87±1.51a	7.87±0.71abc	7.67±1.09abc	7.19±2.10bc	5.32±0.96cd	3.42±3.42d	
Fructose ($g\ kg^{-1}$)	4	11.05±2.86ab	15.33±1.77a	9.89±3.07ab	12.07±3.15ab	9.71±2.84ab	14.07±1.75ab	10.71±2.13ab	11.03±1.98ab
	13	13.98±1.03ab	14.25±0.92a	8.37±2.92cdef	13.89±0.90ab	10.40±3.03bc	8.96±2.51cd	8.67±0.79cde	9.07±1.66cd
	25	12.36±1.50a	12.55±2.43a	8.72±2.06ab	9.82±2.08ab	8.95±2.98ab	6.27±1.67bc	3.91±1.72c	
Sucrose ($g\ kg^{-1}$)	4	92.27±9.03a	78.79±9.23abc	83.18±9.02ab	77.44±4.06abcd	72.20±11.85abcd	57.29±5.84cde	72.83±11.49abcd	58.29±5.07de
	13	83.02±13.54a	66.35±14.33ab	73.63±10.44ab	54.80±8.93bc	44.90±16.77cd	42.16±8.85cd	29.89±4.31de	27.93±8.56cde
	25	85.18±12.27a	57.00±7.54b	57.25±7.58b	29.14±16.32c	20.17±9.32c	22.74±4.48c	10.35±3.21c	

Table S1 (continue)

Quality attributes	T (°C)	Storage time (day)						
		17	19	21	23	25	27	29
Weight loss (%)	4	2.17±0.35bc	2.10±0.41bcd	2.15±0.47bcd	2.94±0.53a	3.25±0.49a	3.26±0.18a	2.66±0.45ab
	13	3.55±0.44bcd	3.99±0.58bcd	4.52±0.89abc	4.44±0.63bc	4.55±0.69ab	4.34±0.41bc	5.64±0.73a
	25							
Total soluble solids (%)	4	16.09±1.74cde	15.76±0.95cde	15.69±2.16cde	15.33±0.91cde	15.55±0.94cde	14.87±0.96de	14.18±1.52e
	13	9.22±0.99efg	9.05±1.56efg	8.60±0.90fgh	7.17±0.93ghi	6.06±0.93hij	5.91±1.47ij	3.52±0.43j
	25							
Dry matter (%)	4	22.31±2.09ab	21.18±2.33ab	21.15±3.41ab	21.30±1.20ab	21.60±1.27ab	21.47±1.57ab	20.24±2.41b
	13	19.22±2.46b	20.93±1.62ab	20.26±3.20ab	19.29±0.98b	20.08±1.41ab	19.10±0.96b	18.39±1.93b
	25							
Hardness (N)	4	52.95±12.43b	62.19±5.54ab	67.59±14.12ab	68.63±5.03ab	68.10±5.95ab	62.34±2.64ab	61.28±7.33ab
	13	89.91±11.10abc	94.76±8.35abc	92.78±15.39abc	100.74±4.71ab	114.61±22.99a	102.30±6.95ab	111.53±30.40a
	25							
Glucose ($g\ kg^{-1}$)	4	8.83±1.07a	7.17±1.82a	9.09±2.22a	10.28±2.21a	9.82±2.44a	10.39±2.71a	10.39±2.53a
	13	6.79±1.80bcde	5.12±1.70de	6.00±1.78cde	4.23±1.64de	4.70±0.65de	4.21±1.38de	2.81±0.91e
	25							
Fructose ($g\ kg^{-1}$)	4	9.43±1.72ab	8.97±2.82b	10.00±3.33ab	10.47±4.48ab	10.47±2.79ab	10.79±2.44ab	10.16±2.29ab
	13	6.73±1.15cdef	5.93±1.88defg	7.00±1.70cdef	5.75±1.67defg	4.99±1.27efg	4.74±0.90fg	2.73±0.74g
	25							
Sucrose ($g\ kg^{-1}$)	4	60.53±13.98bcde	57.78±12.90cde	54.59±16.49de	53.98±9.64de	54.12±6.59de	47.70±7.41e	43.51±15.66e
	13	21.65±6.14ef	25.64±4.73def	16.92±8.38ef	16.02±7.20ef	11.25±2.54ef	10.95±8.61ef	6.26±2.74f
	25							

Table S2. Estimated kinetic parameters obtained from zero-, first-, and second-order reaction models using the NIR–MCR-ALS concentration profile.

Reaction order	T (°C)	A_0	k	R^{2*}	SL (day)
Zero-	4	19.1983	0.1322	0.6860	41.3
	13	19.2158	0.4976	0.8292	11.0
	25	20.1554	0.7182	0.9864	8.9
First-	4	2.9585	0.0078	0.6860	43.6
	13	3.2408	0.0643	0.5898	9.6
	25	3.0386	0.0495	0.9681	8.4
Second-	4	0.0517	-0.0005	0.6844	46.2
	13	-0.0599	-0.0146	0.4012	9.1
	25	0.0450	-0.0035	0.9393	7.9

R^{2*} = coefficient of determination of the kinetic degradation model; SL = predicted shelf life.

Table S3. Activation energy constants (E_a) of the Arrhenius equation and shelf-life predictions from NIR–MCR-ALS concentration profile using the zero, first and second-order reactions.

Reaction order	T (°C)	k (day^{-1})	$\alpha_{T,4}$	E_a (kJ mol^{-1})	R^{2*}	Cut-off	SL (day)	Relative error (%)
Zero-	4	0.1322	1.0000	54.05	0.8387	13.74	48.5	-
	13	0.4976	3.7637				12.9	-
	25	0.7182	5.4330				8.9	1.1
First-	4	0.0078	1.0000	57.47	0.2377	2.62	53.6	-
	13	0.0643	8.2958				6.5	-
	25	0.0495	6.3820				8.4	6.1
Second-	4	-0.0005	1.0000	57.84	0.0064	0.07	55.5	-
	13	-0.0146	32.0190				1.9	-
	25	-0.0035	7.6588				7.9	11.8

R^{2*} = coefficient of determination of the Arrhenius model; $\alpha_{T,4}$ = acceleration factor relative to the reference temperature (4 °C); SL = predicted shelf life.

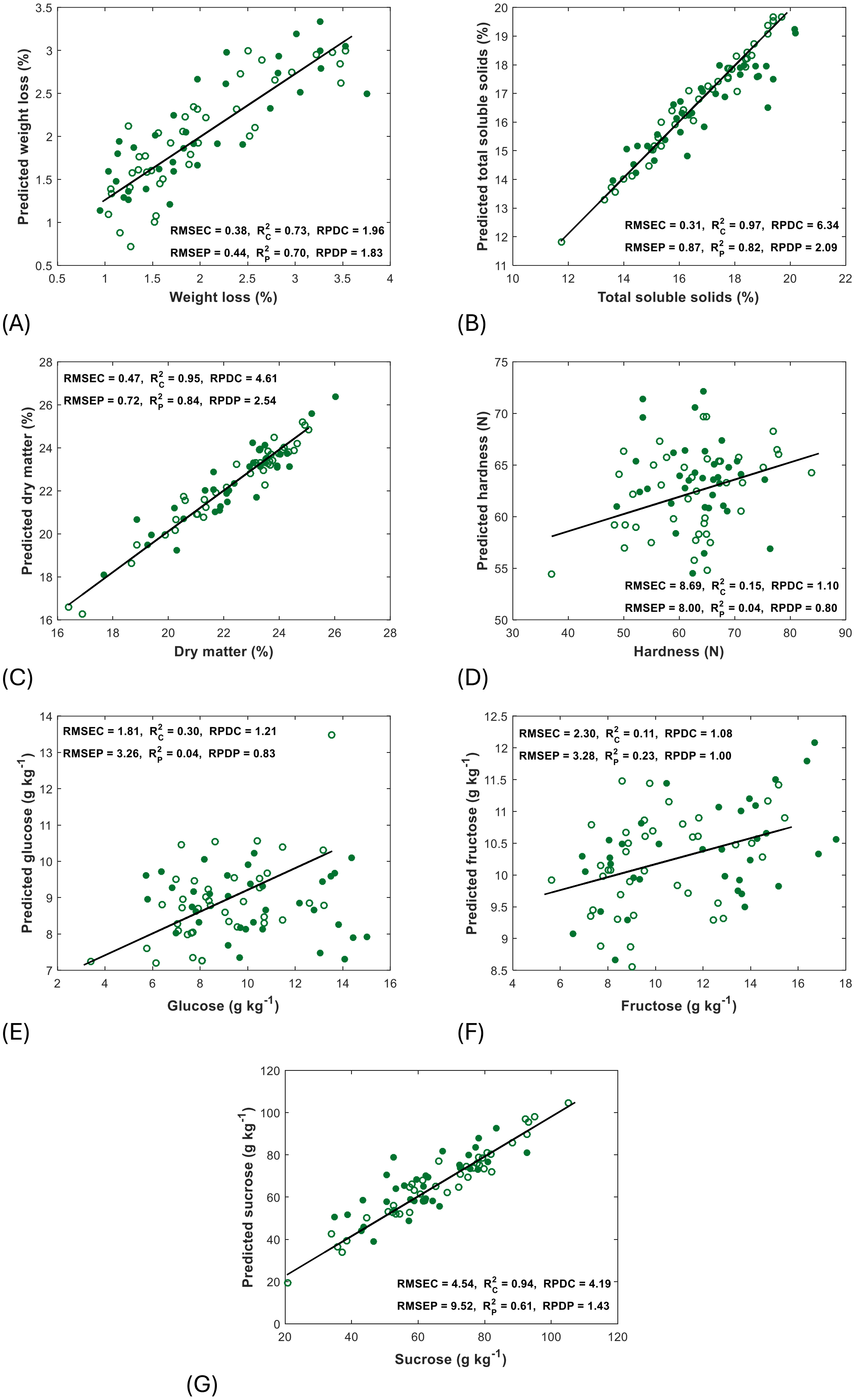

Figure S1. Correlation plots between measured and predicted quality parameters based on NIR spectral data of training and test samples (open- and closed-circle symbols, respectively) stored at 4 °C using PLS.

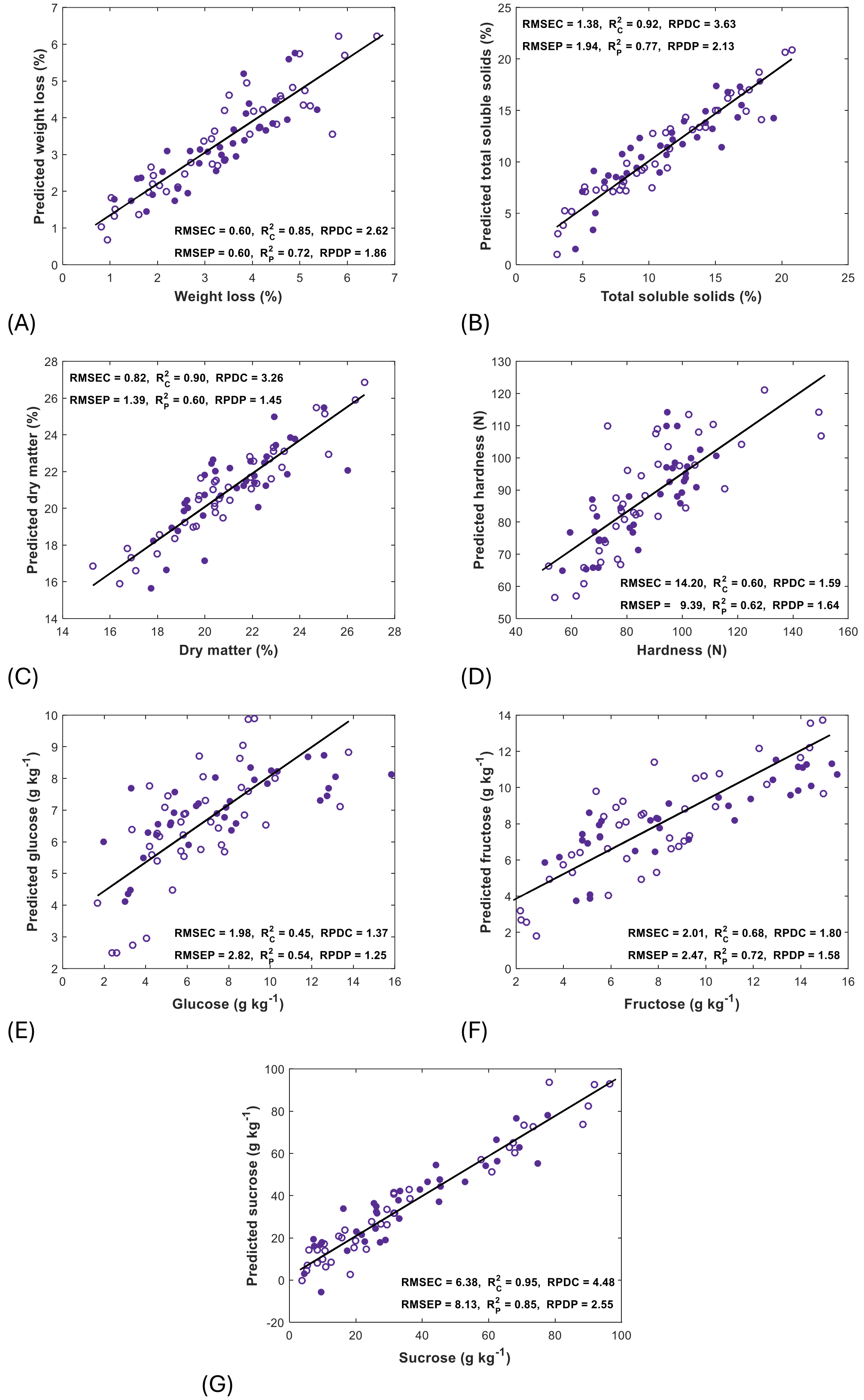

Figure S2. Correlation plots between measured and predicted quality parameters based on NIR spectral data of training and test samples (open- and closed-circle symbols, respectively) stored at 13 °C using PLS.

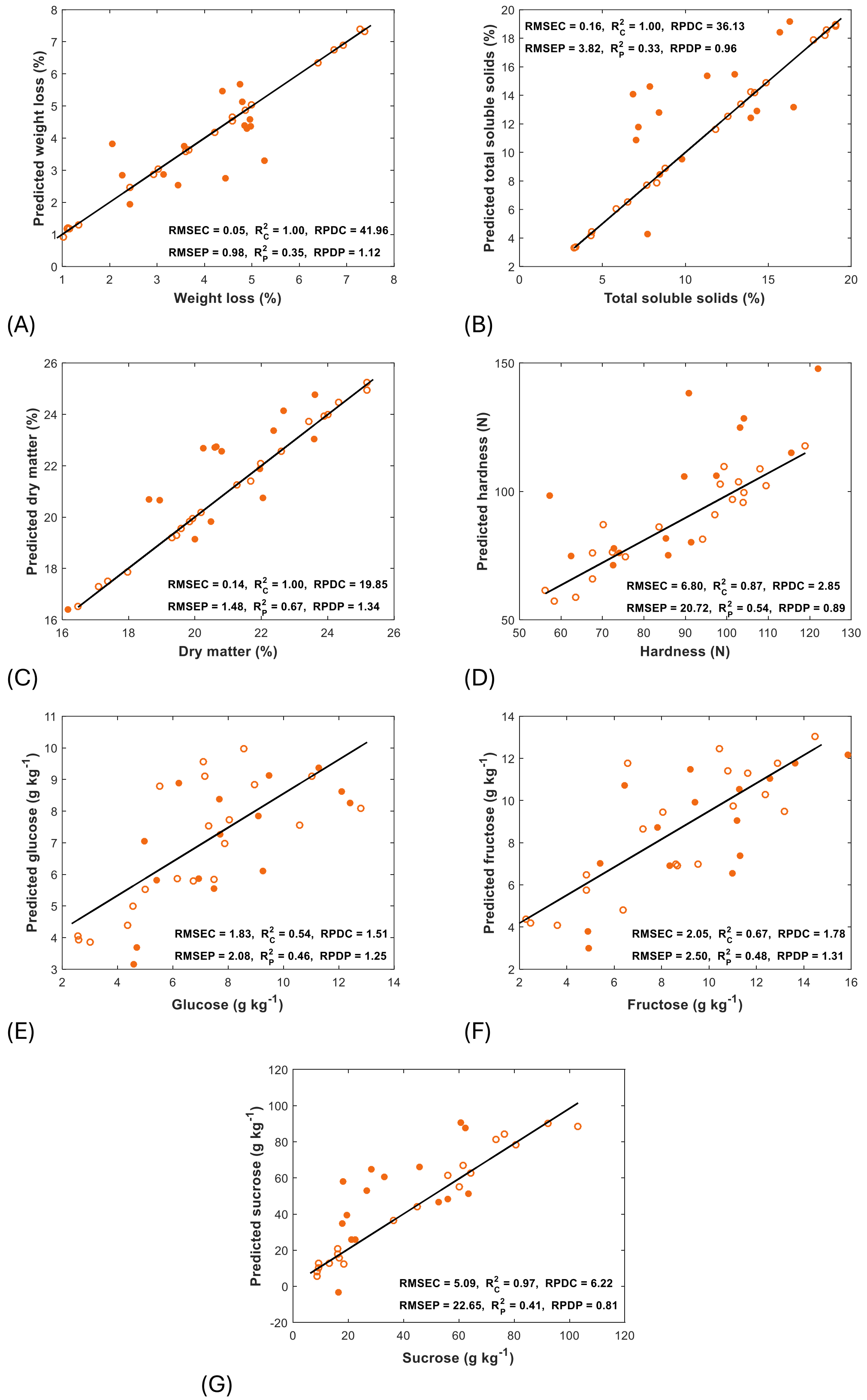

Figure S3. Correlation plots between measured and predicted quality parameters based on NIR spectral data of training and test samples (open- and closed-circle symbols, respectively) stored at 25 °C using PLS.
